# Human T Lymphocyte Isolation, Culture and Analysis of Migration *In Vitro*

**DOI:** 10.3791/2017

**Published:** 2010-06-01

**Authors:** Craig T. Lefort, Minsoo Kim

**Affiliations:** Center for Vaccine Biology and Immunology, University of Rochester

## Abstract

The migration of T lymphocytes involves the adhesive interaction of cell surface integrins with ligands expressed on other cells or with extracellular matrix proteins. The precise spatiotemporal activation of integrins from a low affinity state to a high affinity state at the cell leading edge is important for T lymphocyte migration ^1^. Likewise, retraction of the cell trailing edge, or uropod, is a necessary step in maintaining persistent integrin-dependent T lymphocyte motility ^2^. Many therapeutic approaches to autoimmune or inflammatory diseases target integrins as a means to inhibit the excessive recruitment and migration of leukocytes ^3^.  To study the molecular events that regulate human T lymphocyte migration, we have utilized an *in vitro* system to analyze cell migration on a two-dimensional substrate that mimics the environment that a T lymphocyte encounters during recruitment from the vasculature. T lymphocytes are first isolated from human donors and are then stimulated and cultured for seven to ten days. During the assay, T lymphocytes are allowed to adhere and migrate on a substrate coated with intercellular adhesion molecule-1 (ICAM-1), a ligand for integrin LFA-1, and stromal cell-derived factor-1 (SDF-1). Our data show that T lymphocytes exhibit a migratory velocity of ~15 μm/min. T lymphocyte migration can be inhibited by integrin blockade ^1^ or by inhibitors of the cellular actomyosin machinery that regulates cell migration ^2^.

**Figure:**
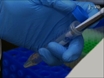


## Protocol

### 1. Isolation of Human T Lymphocytes

1. Obtain human blood from a healthy donor. Allow the blood to cool to room temperature (~30 min) before proceeding to the next step.
2. Gently pipette 3 mL of room temperature Polymorph density gradient media into an 8 mL round-bottom polystyrene tube. Gently add 3 mL of whole blood on top of the Polymorph media. It is important to avoid mixing of the two reagents.
3. Centrifuge the tubes at 500 x g for 45 minutes at room temperature.
4. Following the centrifugation, the peripheral blood mononuclear cells (PBMC) have now separated from other blood components into the top cell layer. The PBMC layer appears, from the top down, as the first cloudy band.
5. Carefully remove the clear yellow-colored upper phase of the blood, above the PBMC layer, and then use a P1000 micropipette to transfer the PBMC layer to a 15 mL or 50 mL conical tube.
6. Wash the PBMC twice with PBS, centrifuging cells at 500 x g for 5 minutes each time. The supernatant will be somewhat cloudy after each wash.

### 2. Culture of Human T Lymphocytes

1. Using a pipette, transfer the PBMC to a T-75 culture flask in 20 mL RPMI 1640 media containing 10% FBS, 1% penicillin/streptomycin, and 1 μg/mL phytohemagglutinin (PHA).
2. Incubate at 37°C and 5% CO_2_ for at least 1 hour, and up to 24 hours. This step allows monocytes, which will be adherent to the flask surface, to be separated from the lymphocytes that remain in suspension. If a short incubation (1 hour) is used at this step, it is acceptable to use RPMI 1640 media containing 10% FBS and 1% penicillin/streptomycin without supplementing with PHA as specified in step 2.1.
3. Carefully remove all of the media from the flask, add it to a 50 mL conical tube, and centrifuge at 500 x g for 5 minutes.
4. Resuspend the cell pellet, which now primarily contains lymphocytes, and transfer the cells to a new T-75 flask containing 25 mL RPMI 1640 media containing 10% FBS, 1% penicillin/streptomycin, and 1 μg/mL PHA.
5. Incubate at 37°C for 3 days (2 days if the initial incubation of PBMC was overnight). After 24 hours of growth, it may be necessary to add 15-20 mL of fresh media and transfer to a larger T-175 flask.
6. After 3 days, use a pipette to remove the media and suspended lymphocytes from the flask and transfer to a 50 mL conical tube. Centrifuge at 500 x g for 5 minutes.
7. Resuspend the cell pellet and transfer cells to a new T-75 or T-175 flask containing 25 mL (T-75) or 50 mL (T-175) RPMI 1640 with 10% FBS, 1% penicillin/streptomycin, and 20 ng/mL human IL-2 or IL-15.
8. Grow lymphocytes for 4-7 days. If starting with a T-75 flask, the culture will need to be expanded and transferred to a T-175 flask after 1-2 days.

### 3. *In Vitro* Lymphocyte Migration Assay

1. 1 day before the migration assay, coat a glass-bottom 0.17 mm dish with 20 μg/mL Protein A or G in PBS overnight at 4°C.
2. Wash the dish extensively with PBS.
3. Immobilize human ICAM-1/Fc (10 μg/mL) and human SDF-1 (2 μg/mL) by adding these reagents in PBS solution to the dish and incubating 4 hours at room temperature.
4. Prepare T lymphocytes by first determining the cell density in culture in the T-175 flask using a hemacytometer. Approximately 2-5 x 10^5^ cells should be used per dish to achieve a cell density appropriate for migration analysis.
5. Wash T lymphocytes twice with PBS and then resuspend in 1 mL L-15 media containing 1 mg/mL D-glucose.
6. Wash the dish coated with ICAM-1/Fc and SDF-1 extensively with PBS.
7. Transfer T lymphocytes in 1 mL media to the dish and maintain them at 37°C.

### 4. Capturing an Image Sequence Using NIS Elements Software

1. Open NIS Elements software.
2. In the camera settings menu, choose "2x2 binning" as the mode for both live imaging and image capture.
3. Go to the Applications menu and choose Define/Run Experiment.
4. Choose the length of time between images and the total length of the image capture sequence.
5. Press the "Run" button to begin image acquisition.
6. Cells can be tracked and migration parameters (velocity, path length, displacement, etc.) quantified using one of several software packages, including ImageJ, AutoQuant or Volocity (Figure 1).
7. To generate a "spider web plot," the x-y coordinates for each cell and timepoint are collected (Figure 2) and projected onto a graph with a common starting point for each cell at the origin (Figure 3).

### 5. Representative Results

On day 6 of culture in the presence of either IL-2 or IL-15, T lymphocytes make up >98% of cells, as determined by positive CD3 staining as well as positive CD4 and/or CD8 staining. For IL-2, we found 83% CD4+ cells, 15% CD8+ and <1% CD4+CD8+ cells. For IL-15, we found 88% CD4+ cells, 11% CD8+ and <1% CD4+CD8+ cells. During T lymphocyte migration on ICAM-1/SDF-1 substrates, cells exhibited a velocity of approximately 15 μm/min that can be sustained over a 1 hour time period. T lymphocyte migration on ICAM-1/SDF-1 is dependent on LFA-1-mediated adhesion, as an anti-LFA-1 ligand-blocking antibody severely inhibits migration.


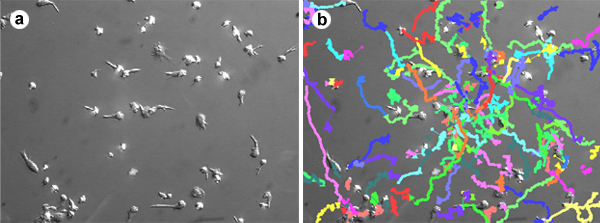
 **Figure 1. Cell tracking of migrating T lymphocytes.** T lymphocytes isolated from whole blood and cultured in the presence of IL-2 or IL-15 for 6 days were allowed to adhere and migrate on glass-bottomed dishes coated with 10 μg/mL ICAM-1 and 2 μg/mL SDF-1. Images were acquired every 10 seconds for 30 minutes. Cells were identified in each image and tracked over time using Volocity software. This movie shows the random migration of T lymphocytes at a velocity of ~15 μm/min.

Click here to see the video. **Movie 1.** Cell tracking of migrating T lymphocytes. T lymphocytes isolated from whole blood and cultured in the presence of IL-2 or IL-15 for 6 days were allowed to adhere and migrate on glass-bottomed dishes coated with 10 μg/mL ICAM-1 and 2 μg/mL SDF-1. Images were acquired every 10 seconds for 30 minutes. Cells were identified in each image and tracked over time using Volocity software. This movie shows the random migration of T lymphocytes at a velocity of ~15 μm/min.


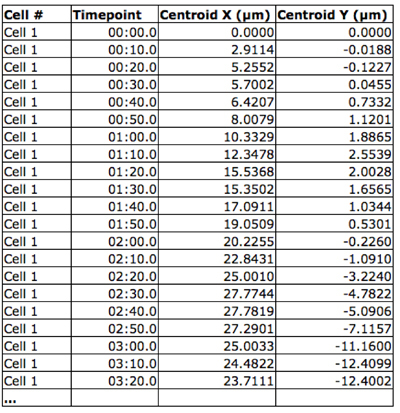
 **Figure 2.** Spatiotemporal cell position. The X-Y coordinates of each cell were obtained for each time point using Volocity software.


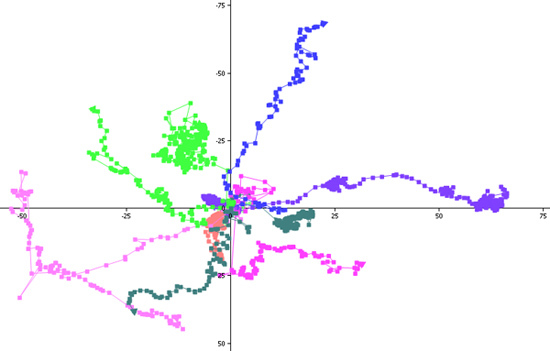
 **Figure 3.** "Spider web plot." Using the X-Y-t coordinates for each cell, 15 cells were chosen at random and plotted with a common starting point at the origin. Comparing spider web plots gives a quick visual depiction of differences in migration between different experimental conditions. Plots for T lymphocyte migration under control conditions (left panel) and in the presence of an anti-LFA-1 ligand-blocking antibody (right panel).

## Disclosures

No conflicts of interest declared.

## Discussion

In this experiment, we provide details on a simple system to analyze the motility of primary human T lymphocytes. *In vitro* migration assays have been used to dissect the roles of many molecules and signaling pathways involved in the locomotion of various cell types. Some critical controls to keep in mind when designing your own experiment from our protocol include: 1) Non-adhesive or non-integrin adhesive substrate coatings, such as bovine serum albumin (BSA) or poly-L lysine (PLL), respectively, 2) integrin-specific blocking antibody treatment to determine integrin specificity, and 3) co-coating without stimulatory signal, such as SDF-1 described in our protocol.
